# Association of IL-1A and IL-1B polymorphisms with ankylosing spondylitis among the Chinese Han population: a case–control study

**DOI:** 10.18632/oncotarget.16004

**Published:** 2017-03-08

**Authors:** Lei Li, Baolan Shi, Wenkai Zheng, Wenhua Xing, Yan Zhao, Feng Li, Daqi Xin, Tianbo Jin, Yong Zhu, Xuejun Yang

**Affiliations:** ^1^ Inner Mongolia Medical University, Hohhot 010020, China; ^2^ The Second Affiliated Hospital of Inner Mongolia Medical University, Hohhot 010030, China; ^3^ Key Laboratory of Resource Biology and Biotechnology in Western China (Northwest University), Ministry of Education, Xi’an, Shaanxi 710069, China; ^4^ Inner Mongolia Medical University, Chifeng Clinical Medical College, ChiFeng, 024000, China

**Keywords:** ankylosing spondylitis (AS), single nucleotide polymorphism (SNP), IL-1A, IL-1B, case-control study

## Abstract

Ankylosing spondylitis (AS) is a complex and chronic inflammatory disease with a high heritage. Previous study has shown that IL-1A and IL-1B involved in inflammatory reaction. But little is known about single nucleotide polymorphisms (SNPs) of IL-1A and IL-1B associated with AS. We conducted a case-control study among 267 AS cases and 297 healthy controls from China. In the genetic model analysis, we found the “T” genotype of rs3783550 was associated with decreased AS risk in the dominant model (*p* = 0.044) and log-additive model (*p* = 0.023); the “C” genotype of rs3783546 was significantly associated with decreased AS risk based in the dominant model (*p* = 0.044) and log-additive model (*p* = 0.023). Additionally, the minor allele “A” of rs2853550 may also reduce the risk of AS in dominant (*p* = 0.025) and log-additive model (*p* = 0.024). Our results suggested that the polymorphisms of IL-1A and IL-1B are associated with the AS susceptibility in the Chinese Han population. Further studies are needed to characterize the functional sequences that cause AS.

## INTRODUCTION

Ankylosing spondylitis (AS) is a complex and chronic inflammatory disease with a high heritage. The overall prevalence of AS in Chinese is 0.2–0.54% [1]. The condition affects predominantly the axial skeleton, including the spine and sacroiliac joints, and causes pain, stiffness, and, eventually, bony ankylosis. Joints and tendon insertions (entheses) elsewhere are also commonly involved, and approximately one-third of patients develop acute anterior uveitis.

Although AS is also a complex multifactorial disease resulting from gene and environment interaction, the exact mechanism of AS is still unclear. As we all know, Genetic factors have an importance role in AS susceptibility and expression [2]. HLA–B27 is the major gene associated with the susceptibility of AS, which is present in > 95% of Caucasians of northern European ancestry, yet only 1%–5% of HLA-B27 carriers develop AS, so that HLA-B27 carriage alone does not explain the pattern of disease recurrence in families [3]. Therefore, we provide a hypothesis that HLA-B27 is almost essential but not adequate to cause AS and that other genes interact with HLA-B27 to cause the condition. Recent genome-wide screening identified non-MHC regions with linkage with AS [4, 5]. The interleukin-1 (*IL-1*) cluster on chromosome 2q has emerged as a robust non-MHC susceptibility locus for AS in several groups study [5–7]. Those studies, however, always have conflicting results in different populations, making it difficult to put forward a conclusion about the relationship between *IL-1* gene cluster and AS. Due to the *IL-1* gene cluster also play a prominent role in inflammation and host defense against infection. The prototypic members of the *IL-1* family gene cluster are the genes *IL-1A*, *IL-1B*, and *IL-1RN* [5], which were related to AS, but *IL-1A* and *IL-1B* were less reported [4, 5, 8, 9]. *IL-1A* and *-B* encode proinflammatory cytokines involved in host defense against infection [5]. The genes polymorphisms have been related to a number of chronic inflammatory diseases such as Crohn's disease and inflammatory bowel disease (IBD) [10, 11]. Rs16944 in *IL-1B* was identified associated with AS in European ancestry [5], but was not replicated in another study in a Han Chinese population, and few information is found about other SNPs on IL-1A and L-1B associated with AS risk. Besides, recent report has showed that association particularly with haplotypes of single nucleotide polymorphisms (SNPs) in the IL1RN gene [4], suggesting that the true associated polymorphism lay on the associated haplotype but was not one of the individual markers genotype.

Thus, in consideration of these circumstances, we sought to test whether *IL-1* genes were associated with AS in Chinese Han population, which is previously associated with AS in Europe population.

## RESULTS

A total of 276 cases and 296 controls were enrolled in our study. The demographic characteristics of the study population are showed in Table 1, which showed the significant difference in age and gender distribution between the case and control groups (*p* < 0.001).

**Table 1:** Number of individuals for ankylosing spondylitis case and controls

Variables Case (*n* = 267)	Control (*n* = 297)	Total	P*
Sex < 0.001^a^			
Female	67 (25.1%)	198 (66.7%)	265
Male	200 (74.9%)	99 (33.3%)	299
Age, yr (mean ± SD)	31.58 ± 12.28	56.35 ± 9.72	<0.001^b^

^a^Pearson^’^s test;

^b^Welch^’^s test.

Table 2 summarized the minor allelic frequency (MAF) of tested SNPs among the individuals in the case and control groups. After Hardy–Weinberg equilibrium (HWE) *p* value screening, we excluded one SNP (rs2853550). The allelic frequency of *SNPs* in the controls group was similar to those of the HapMap Asian population. Through the χ^2^ test, we found that no SNP was significantly associated with AS risk.

**Table 2:** Allele frequencies in cases and controls and odds ratio estimates for ankylosing spondylitis

SNP ID	Genes	Role	AllelsAa/B	HWE *P*-value	MAF		ORs	95 % CI	*P*^b^
					Case	Control			
rs3783550	*IL1A*	Intron	T/G	0.790	0.337	0.320	1.08	0.84–1.39	0.539
rs3783546	*IL1A*	Intron	C/G	0.894	0.337	0.319	1.08	0.85–1.39	0.523
rs2856838	*IL1A*	Intron	A/G	0.748	0.253	0.235	1.10	0.84–1.45	0.482
rs1609682	*IL1A*	Intron	T/G	0.789	0.327	0.321	1.08	0.84–1.38	0.564
rs3783521	*IL1A*	Promoter	G/A	0.790	0.306	0.320	1.08	0.84–1.39	0.539
rs2853550	*IL1B*	Downstream	A/G	0.032^#^	0.082	0.082	1.01	0.66–1.55	0.963
rs1143643	*IL1B*	Intron	C/T	0.350	0.444	0.475	0.88	0.70–1.12	0.302
rrs3136558	*IL1B*	Intron	G/A	1.000	0.380	0.380	1.00	0.79–1.27	0.998
rs1143630	*IL1B*	Intron	T/G	0.654	0.180	0.153	1.21	0.88–1.66	0.231
rs1143627	*IL1B*	Promoter	G/A	0.412	0.466	0.486	0.92	0.73–1.17	0.500
rs16944	*IL1B*	Promoter	A/G	0.416	0.470	0.478	0.97	0.77–1.22	0.787
rs1143623	*IL1B*	Promoter	G/C	0.398	0.399	0.408	0.96	0.76–1.22	0.762

HWE: Hardy-Weinberg equilibrium; MAF: Minor allelic frequency

OR: odds ratio; 95%CI: 95%confidence interval;

SNP: Single-nucleotide polymorphism;

^a^Minor allele;

^b^*p* ≤ 0.05 indicates statistical significance;

^#^ HWE *p* ≤ 0.05 was excluded.

The minor allele of each SNP was assumed a risk allele compared to the wild-type allele. MAF in cases and controls are listed in Table 3. Five models (co-dominant model, over-dominant model, log-additive model additive model, dominant model and recessive model) were applied for analyzing the association between polymorphisms and AS, which was modified by the age of the subjects. We found that the risk allele “T” of rs3783550 was associated with a decreased risk of AS based on dominant model (OR = 0.56, 95% CI = 0.32–0.99, *p* = 0.044), and log-additive model (OR = 0.61, 95% CI = 0.39–0.94, *p* = 0.023). The minor allele “C” of rs3783546 was associated with a decreased AS risk under dominant model (OR = 0.56, 95% CI = 0.32–0.99, *p* = 0.044), and log-additive model (OR = 0.61, 95% CI = 0.39–0.94, *p* = 0.023). Additionally, the minor allele “A” of rs2853550 may also reduce the risk of *AS*, based on the dominant (OR = 0.36, 95% CI = 0.15–0.91, *p* = 0.025) and log-additive model (OR = 0.41, 95% CI = 0.18–0.91, *p* = 0.024). However, no statistically significant evidence suggested that the other polymorphisms tested were associated with AS risk.

**Table 3:** Logistic regression analysis of the association between the SNPs and ankylosing spondylitis disease (adjusted sex and age)

SNP	Model	Genotype	Case	Control	OR (95% CI)	p*	AIC	BIC
rs3783550	Codominant	G/G	136 (45.8%)	117 (43.8%)	1.00	0.073	323.2	344.8
		G/T	132 (44.4%)	120 (44.9%)	0.63 (0.34–1.14)			
		T/T	29 (9.8%)	30 (11.2%)	0.35 (0.13–0.99)			
	Dominant	G/G	136 (45.8%)	117 (43.8%)	1.00	0.044*	322.3	339.7
		G/T-T/T	161 (54.2%)	150 (56.2%)	0.56 (0.32–0.99)			
	Recessive	G/G-G/T	268 (90.2%)	237 (88.8%)	1.00	0.090	323.5	340.8
		T/T	29 (9.8%)	30 (11.2%)	0.43 (0.16–1.17)			
	Overdominant	G/G-T/T	165 (55.6%)	147 (55.1%)	1.00	0.300	325.3	342.7
		G/T	132 (44.4%)	120 (44.9%)	0.74 (0.41–1.32)			
	Log-additive —		—	—	0.61 (0.39–0.94)	0.023*	321.2	338.5
rs3783546	Codominant	G/G	136 (46%)	117 (43.8%)	1.00	0.074	323.1	344.8
		G/C	131 (44.3%)	120 (44.9%)	0.63 (0.34–1.14)			
		C/C	29 (9.8%)	30 (11.2%)	0.35 (0.13–0.99)			
	Dominant	G/G	136 (46%)	117 (43.8%)	1.00	0.044*	322.3	339.6
		G/C-C/C	160 (54%)	150 (56.2%)	0.56 (0.32–0.99)			
	Recessive	G/G-G/C	267 (90.2%)	237 (88.8%)	1.00	0.090	323.5	340.8
		C/C	29 (9.8%)	30 (11.2%)	0.43 (0.16–1.17)			
	Overdominant	G/G-C/C	165 (55.7%)	147 (55.1%)	1.00	0.310	325.3	342.6
		G/C	131 (44.3%)	120 (44.9%)	0.74 (0.41–1.32)			
	Log-additive —		—	—	0.61 (0.39–0.94)	0.023*	321.2	338.5
rs2853550	Codominant	G/G	251 (85.4%)	227 (85%)	1.00	0.076	322	343.6
		A/G	38 (12.9%)	36 (13.5%)	0.38 (0.15–1.00)			
		A/A	5 (1.7%)	4 (1.5%)	0.22 (0.02–3.31)			
	Dominant	G/G	251 (85.4%)	227 (85%)	1.00	0.025*	320.1	337.4
		A/G-G/G	43 (14.6%)	40 (15%)	0.36 (0.15–0.91)			
	Recessive	G/G-A/G	289 (98.3%)	263 (98.5%)	1.00	0.290	324	341.3
		A/A	5 (1.7%)	4 (1.5%)	0.26 (0.02–3.61)			
	Overdominant	G/G-A/A	256 (87.1%)	231 (86.5%)	1.00	0.050	321.3	338.6
		A/G	38 (12.9%)	36 (13.5%)	0.40 (0.15–1.02)			
	Log-additive —		—	—	0.41 (0.18–0.91)	0.024*	320	337.4

AIC: Akaike's Information criterion;

BIC: Bayesian Information criterion;

*p value ≤ 0.05 indicates statistical signifcance.

**Figure 1:**
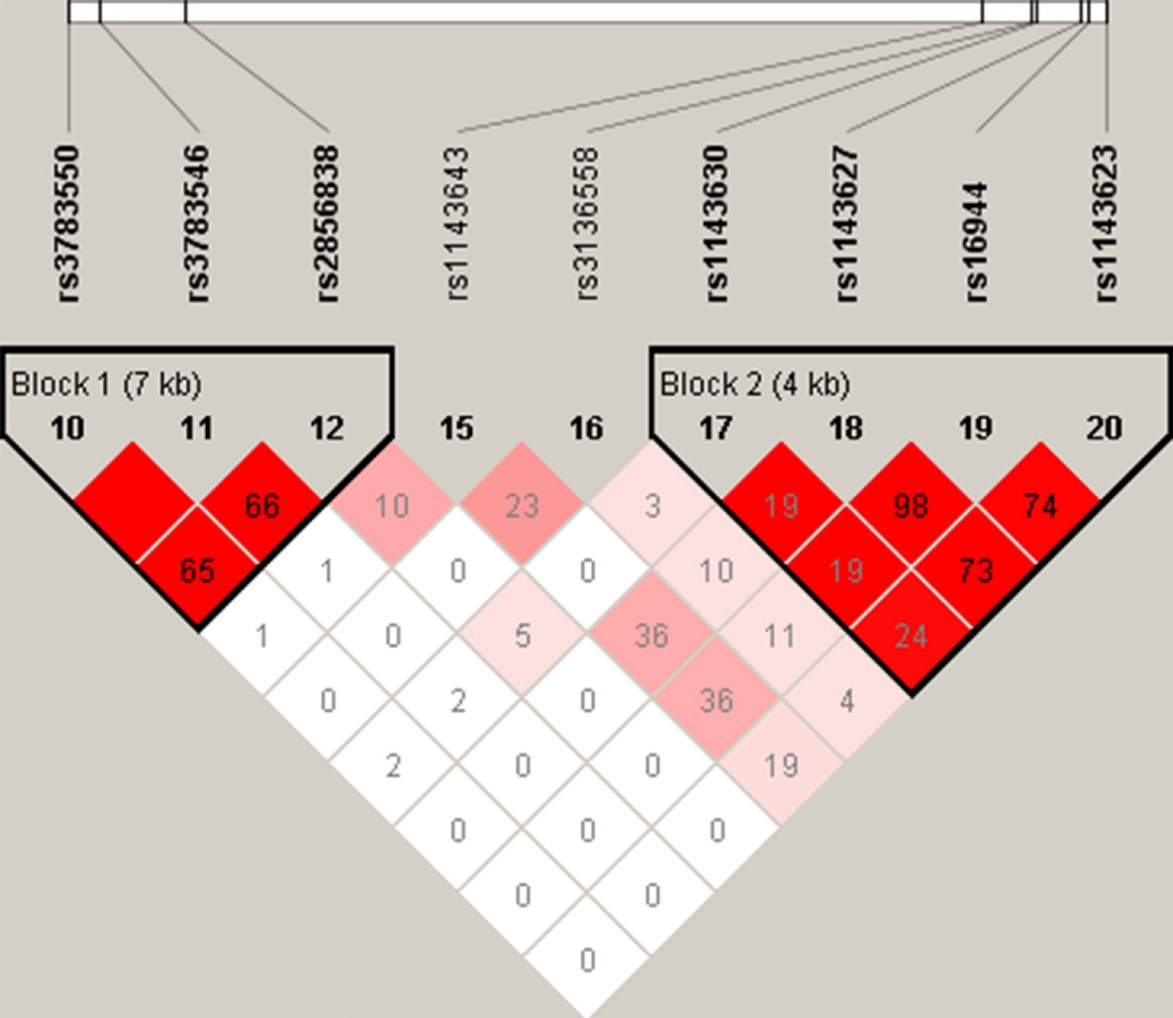
LD analysis of the association between all the SNPs of the IL-1A and IL-1B gene and ankylosing spondylitis

Two blocks were detected in studied *IL-1A* and *IL-1B* SNPs by haplotype analyses (Figure 1). The results of the association between the *IL-1A* and *IL-1B* haplotype andthe risk of AS were listed in Table 4. Haplotype “TCG” of *IL-1A* in Block were found to be associated with decrease risk of AS (OR = 0.36, 95% CI = 0.16–0.81 *p* = 0.014).

**Table 4:** IL-1A and IL-1B haplotype frequencies and the association with the risk of ankylosing spondylitis in case and control patients (adjusted sex and age)

Gene	SNPs	Haplotype	Freq (case)	Freq(control)	OR(95% CI)	*P*-value*
IL-1A	rs3783550/rs3783546/rs2856838	GGG	0.662	0.680	1(referent)	—
		TCA	0.252	0.237	0.72(0.44–1.18)	0.19
		TCG	0.086	0.083	0.36(0.16–0.81)	0.014*
IL-1B	rs1143630/rs1143627/rs16944/rs1143623	CAGC	0.530	0.517	1(referent)	—
		GGAG	0.223	0.255	1.08(0.68–1.73)	0.74
		TGAG	0.178	0.151	0.89(0.52–1.52)	0.67
		GGAC	0.066	0.070	0.68(0.30–1.56)	0.37

*p value ≤ 0.05 indicates statistical signifcance.

## DISCUSSION

In this Chinese Han population-based case–control study, we investigated the associations between the 12 SNPs and risk of AS. We found the associations between *IL-1A* and *IL-1B* SNPs and the risk of AS, rs3783550, rs3783546 and rs2853550 showed a protective effect on AS risk.

The haplotype “TCG” of *IL-1A* were significance associated with a decreased risk of AS, which can act as a protect factor for AS. Although the individual polymorphism investigated showed no association with AS adjusted by age and sex, it is contribute to our hypothesis that substantially stronger haplotype associations with SNPs of *IL-1A* than with individual SNPs.

IL-1A and IL-1B are one of the family of IL-1 and are proinflammatory cytokine that are mainly produced by activated macrophages, whose actions include augmentation of activation of T and B lymphocytes and monocyte/macrophages, and induce fibroblast proliferation leading to synovial pannus formation [12], which also produce IL-1α and IL-1β, respectively [13, 14]. The genes for the proinflammatory cytokines IL-1α and IL-1β are located on the long arm of human chromosome 2, in close linkage with another gene of the *IL-1* gene family, the gene encoding their endogenous antagonist IL-lRa [15]. Randomized clinical trials in rheumatoid arthritis with recombinant human IL-1ra demonstrated modest anti-inflammatory effects and significant slowing in the rate of joint damage [16]. This may suggest that in certain patients the lack of production of IL-1ra, the natural antagonist of IL-1β, contributes to the chronicity of the inflammation. Furthermore, *IL1A* and *IL-1B* may induce inflammation in AS through intracellular effects [17], it is also possible that *IL1A* and *IL-1B* are involved in the initiation of disease, but has a less significant role in established disease.

A previous study found that rs3783550 was significantly associated with reduced AS risk in Canadian populations [8]. Rs3783546 and rs2853550 were associated with erythematosus [18] and rheumatoid arthritis [19], respectively. So that we speculated that rs3783550, rs3783546 and rs2853550 play an important role in chronic inflammatory disease, but three SNPs were not significantly associated with *AS* risk, only were associated with *AS* in different model in our study. That result might be due to differences among different regions of the world, which needs us to do further research to verify the results.

Despite this study showed three SNPs (rs3783550, rs3783546 andrs2853550) associated with AS susceptibility in the different model, some limitations should be considered. First, as the incidence of ankylosing spondylitis is low, so that the sample size of our study was relatively small. There may be some false positive results, but it has been able to explain the trend of IL-1A and IL-1B in patients with ankylosing spondylitis. In the future we will recruit more samples included in our future studies to verify our results. Second, the heterogeneity in living conditions was not evaluated in this study, which has contributed to the progress in elucidating the pathogenesis of AS.

In conclusion, our study has described the association between rs3783550 (*IL-1A*), rs3783546 (*IL-1A*) and rs2853550 (*IL-1B*) and AS risk and between a new haplotype, “TCG”, of rs3783550, rs3783546 and rs2853550 and AS in Chinese Han population. Therefore, the results provide additional evidence for the relationships between genetic variants and AS susceptibility in the Hainan population. Moreover, a large number of genetic risk factors for AS should be investigated and validated to fully understand the pathogenesis of AS. Further studies will focus on the functional experiments based on the relevant genes on animal models, to investigate detailed mechanism involved.

## MATERIALS AND METHODS

### Study participants

We recruited a total of 267 patients diagnosed with AS and 297 controls from 2014 to 2016 among Han Chinese. All the subjects were treated by the Second Affiliated Hospital of Inner Mongolia Medical University. All participants signed informed written consent. All cases were verified, and patients were recruited without age, or disease stage restriction and who were previously healthy. AS was defined by the modified New York diagnostic criteria [20]. In all cases the diagnosis of AS was confirmed by a qualified rheumatologist and was followed by either examination or telephone interview. In cases with an atypical history or no previous radiographic evidence, pelvic and lumbosacral spine radiographs were obtained and attending general practitioners were contacted to confirm diagnosis. All cases were HLA-B27 positive. To reduce the potential environmental and therapeutic factors impacting the variation of complex human diseases, we performed detailed recruitment and set exclusion criteria to exclude subjects with chronic disease and conditions involving vitalorgans (brain, liver, heart, and lung). Controls were selected unrelated and randomly. Among control individuals, none had any chronic diseases. The use of samples was approved by the Human Research Committee of the Second Affiliated Hospital of Inner Mongolia Medical University for Approval of Research Involving Human Subjects. The personal characteristics of the participants are listed in Table 1.

### SNP selection and genotyping

Among the 12 SNPs we selected, rs16944 in IL-1B [5] was chosen from previously published polymorphisms associated with AS, others were randomly chosen from the published genes (IL-1A and IL-1B) associated with AS. Minor allele frequencies of all SNPs were > 5%, in the HapMap of the Chinese Han CHB population. We adopted Gold Mag-Mini Whole Blood Genomic DNA Purification Kit (GoldMagCo. Ltd. Xi’an City, China) to extract DNA that was from whole blood. Quantification of the extracted DNA was performed using NanoDrop2000. Genotyping was done with the Sequenom MassARRAY RS1000 system using the standard protocol recommended by the manufacturer. The multiplexed SNP Mass EXTENDED assay was designed using Sequenom Mass-ARRAY Assay Design 3.0 Software [21]. Data management and analysis was done using SequenomTyper 4.0 Software. We use the Sequenom MassARRAY RS1000 system to analysis genotype, which was conform to the standard protocol recommended by the manufacturer.

### Statistical analysis

We used Microsoft Excel and SPSS 18.0 (SPSS, Chicago, IL, USA) to perform statistical analyses. In this study, all *p* values were two-sided, and *p* ≤ 0.05 was regarded as getting statistical significance. Control genotype frequencies for each SNP were tested for departure from Hardy-Weinberg equilibrium (*HWE*) using Fisher's exact test. The χ^2^ test was used to compare the distribution of marker alleles and genotypes in cases and controls [22]. Unconditional logistic regression analysis adjustment with age was used to test odds ratios (ORs) and 95% confidence intervals (CIs) [23]. Associations between SNPs and risks of ASwere tested in genetic models by analysis with SNP Stats software, obtained from http://bioinfo.iconcologia.net. Values of OR and 95% CI were calculated as above.Akaike's InformationCriterion (AIC) and Bayesian InformationCriterion (BIC) were applied to choose the best-fit model for each SNP. Finally, we used the SHEsis software (http://analysis.bio-x.cn/myAnalysis.php) to estimate the pairwise linkage disequilibrium (LD) and haplotype construction [24].
